# Effect of bone marrow mononuclear cells therapy on long-term survival of patients with liver cirrhosis: A 10-year retrospective cohort study using propensity score matching

**DOI:** 10.1515/jtim-2025-0044

**Published:** 2025-10-25

**Authors:** Mengfan Ruan, Yuhang Yin, Wen Ning, Beilei Zhang, Hao Lin, Huiying Yu, Xiaoxi Wang, Deli Zou, Xiaodong Shao, Jiang Chen, Hongyu Li, Xiaozhong Guo, Xingshun Qi

**Affiliations:** Department of Gastroenterology, General Hospital of Northern Theater Command (Teaching Hospital of Jinzhou Medical University), Shenyang, Liaoning Province, China; Postgraduate College, China Medical University, Shenyang, Liaoning Province, China; Postgraduate College, Dalian Medical University, Dalian, Liaoning Province, China; Laboratory of Basic Medicine, General Hospital of Northern Theater Command, Shenyang, Liaoning Province, China

**Keywords:** cirrhosis, bone marrow mononuclear cells, survival, follow-up, long-term prognosis

## Abstract

**Background:**

Bone marrow mononuclear cells (BMMNCs) therapy should be effective for the improvement of liver function and short-term outcome in patients with liver cirrhosis, but few studies have explored the long-term prognosis of cirrhotic patients treated with BMMNCs.

**Methods:**

In this retrospective study, eligible patients with liver cirrhosis were selected by using propensity score matching (PSM). Effect of BMMNCs on death was explored by Cox regression analysis, as well as competing risk analysis, where liver transplantation was a competing event. Hazard ratio (HR) and sub-distribution HR (sHR) were calculated. Subgroup analyses were performed based on the age, sex, Child-Pugh class, and model for end-stage liver disease (MELD) score.

**Results:**

Overall, 260 patients were included, of whom 130 were treated with transhepatic arterial transplantation of BMMNCs. The median follow-up duration was 5.27 (range: 0.37–16.62) years. By adjusting by age, sex, and Child-Pugh score, multivariate Cox regression (HR = 0.707, *P* = 0.020) and competing risk analyses (sHR = 0.709, *P* = 0.026) demonstrated that BMMNCs were independently associated with a lower risk of death in cirrhotic patients in the overall analysis. Univariate Cox regression analyses demonstrated that BMMNCs were significantly associated with a decreased risk of death in the subgroup analyses of age ≤50 years (HR= 0.533, P = 0.016), male (HR = 0.626, *P* = 0.010), Child-Pugh class B (HR = 0.638, *P* = 0.026), and MELD score > 12 (HR = 0.483, *P* = 0.002), but not age > 50 years (*P* = 0.097), female (*P* = 0.170), Child-Pugh class A (*P* = 0.309), Child-Pugh class C (*P* = 0.369), or MELD score ≤12 (*P* = 0.096).

**Conclusion:**

BMMNCs can provide additional survival benefits in patients with liver cirrhosis.

## Graphical Abstract

**Figure:**
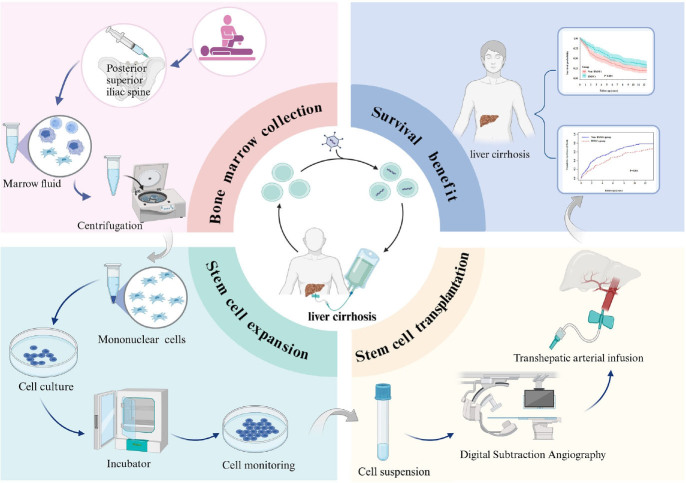

## Introduction

Liver diseases cause about 2 million deaths per year, accounting for 4% of the total deaths globally, primarily due to cirrhosis and its complications.^[1]^ Cirrhosis is associated with long-term inflammation caused by a variety of causes, including viral hepatitis, steatohepatitis, alcohol abuse, and autoimmune hepatitis.^[2]^ Patients with early cirrhosis are often asymptomatic, while those with decompensated cirrhosis can suffer from progressive aggravation of portal hypertension, ascites, hepatic encephalopathy (HE), and liver failure, which compromises the quality of life and survival.^[2, 3, 4]^ When the liver is slightly and transiently damaged, it can be repaired by rapid proliferation of hepatocytes; by comparison, when cirrhosis develops, especially decompensated cirrhosis, liver transplantation is the only curative treatment.^[5, 6, 7, 8, 9, 10]^ However, only a minority of patients who meet the criteria for liver transplantation can undergo liver transplantation due to the lack of liver donors, its high cost, and the requirement of life-long immunosuppressants.^[11]^ Therefore, it is particularly important to seek for other alternatives to repair chronically injured liver and regenerate hepatic parenchymal.^[12]^

Currently, cell therapy has emerged as a promising approach for the treatment of liver-related diseases due to its self-renewal, paracrine, and immunomodulatory effects.^[13,14]^ Bone marrow mononuclear cells (BMMNCs) are a heterogeneous cell population, including hematopoietic stem cells, mesenchymal stem cells, and immune cells. This cell population can produce its therapeutic effect mainly through multiple ways. First, hematopoietic stem cells can differentiate into hepatocyte-like cells to participate in liver tissue repair. Second, mesenchymal stem cells can promote hepatic stellate cell apoptosis and inhibit fibrosis process by secreting hepatocyte growth factor (HGF) and vascular endothelial growth factor (VEGF) and other bioactive factors. Third, immune regulatory cells can reduce liver inflammatory microenvironment.^[15,16]^ Because complex preparation or culture of BMMNCs is not required, they become an attractive option for cell therapy in regenerative medicine. Thus, they have been widely used in experimental and clinical studies.^[17,18]^ Until now, several studies have shown the efficacy of BMMNCs for the treatment of cirrhosis.^[19, 20, 21]^ Notably, current evidence supports the benefits of BMMNCs for improving liver function and short-term prognosis in patients with liver cirrhosis, but their effects on long-term survival have been rarely explored yet.^[22,23]^ Herein, we attempt to address this issue by reviewing the data of cirrhotic patients who were hospitalized at our center and followed for more than 10 years.

## Materials and methods

### Ethics

This study was conducted in accordance with the rules of the Declaration of Helsinki and was approved by the Ethics Committee of the General Hospital of Northern Theater Command with an ethical approval number (Y[2024]208). The patient’s informed consent was not required due to the retrospective nature of this study.

### Study design

By searching the diagnostic code of liver cirrhosis in the electronic medical record system of our hospital, a total of 3799 patients with liver cirrhosis who were consecutively admitted to the Department of Gastroenterology of our hospital from January 1, 2007 to June 30, 2014 were identified. Among them, 270 patients with cirrhosis underwent cells transplantation during hospitalization, who are considered as the BMMNCs group. The exclusion criteria of the BMMNCs group were as follows: (1) repeated admissions of the same patients; (2) patients with a history of liver or other malignancy; (3) patients with incomplete data information related to the study; and (4) the type of cells used for transplantation was not BMMNCs. The remaining 3529 patients with cirrhosis received conventional treatment without cells transplantation during hospitalization, who are considered as the non-BMMNCs group. The exclusion criteria of the non-BMMNCs group were as follows: (1) repeated admissions of the same patients; (2) patients with a history of liver or other malignancy; (3) patients with incomplete data information related to the study; and (4) patients who underwent BMMNCs transplantation at other hospital.

### Propensity score matching analysis

Propensity score matching (PSM) method was used to select the control group from 831 eligible patients with liver cirrhosis. In this study, multivariable logistic regression model was used to estimate propensity scores for all patients with the following baseline characteristics as covariates, including age, gender, Child-Pugh score, and model of end-stage liver disease (MELD) score. According to the propensity score calculated, the one-to-one PSM analysis was performed using the nearest neighbour method with a caliper of 0.2.^[24]^

### Baseline data collection

Demographic, clinical, and laboratory data at admissions were collected, including age, gender, etiology of liver cirrhosis (*i.e*., hepatitis B virus, hepatitis C virus, and alcohol abuse), decompensation event (*i.e*., ascites, HE, and gastrointestinal bleeding), hemoglobin (Hb), white blood cell, platelet count, total bilirubin, albumin (ALB), aspartate aminotransferase, alanine aminotransferase, alkaline phosphatase, serum creatinine, serum sodium, and international normalized ratio. Child-Pugh and MELD scores were calculated.^[25]^

### Groups and definitions

Eligible patients were divided into BMMNCs group and non-BMMNCs group. BMMNCs group was defined as patients with liver cirrhosis who underwent conventional therapy in combination with BMMNCs transplantation, and non-BMMNCs group as patients with liver cirrhosis who received conventional therapy alone. According to the current clinical guidelines, including the European Association for the Study of the Liver and the American Association for the Study of Liver Diseases, conventional treatment strategies of liver cirrhosis and its complications mainly include etiological therapy, antifibrotic drugs, albumin supplementation, diuresis, endoscopic variceal treatment, and other symptomatic treatments.^[26, 27]^

### Procedures

#### Collection and isolation of BMMNCs

After patients signed written informed consents, subcutaneous injection of recombinant human granulocyte colony-stimulating factor (G-CSF) was given at a dosage of 100 μg as bone marrow mobilization for 2 consecutive days before BMMNCs collection. At the day of BMMNCs collection, they were placed in a sterile room, and their posterior superior iliac spines were selected as the point of bone marrow puncture. After local sterilization and anesthesia, 150–200 mL of bone marrow was extracted by an 18-gauge bone marrow puncture needle. Then, remove bone fragments and fat particles through a 70–200 μm filter, add unfractionated heparin, and store at 4 ℃ for less than 24 h. Separate cell components by density gradient. Dilute bone marrow blood with PBS at a ratio of 1: 1–1: 2. Then, they were carefully superimposed on Ficol-Paque (density 1.077 g/mL), centrifuged (400–450 *g*, 30–40 min, room temperature, no brake) to collect mononuclear cell layers, and washed with PBS 2–3 times (300 *g*, 10 min). Isolated cells were resuspended with saline. After that, the number of cells was counted using Trypan blue staining or automatic cell counter. The target number of cells was usually 2–4×10^8^ MNC/ kg body weight. Cell viability was measured using Trypan blue rejection (> 90% viability) or 7-AAD/Annexin V flow assay. Cell quality was strictly controlled, as follows: the microbial test of aerobic bacteria, anaerobic bacteria, and fungi was negative, the endotoxin test was < 5EU/kg, and the proportion and number of CD34^+^ cells in the cells were required to reach the predetermined target dose. The cell separation and purification took about 2.5–3 h. Then, according to the clinical requirements, the cells were fully mixed with 1–5 mL of normal saline in a cell transfer tube, the total volume was about 13–15 mL per patient, and 2 mL was reserved at the Department of Medical Laboratory of our hospital for subsequent quality monitoring. The cell transfer tube was marked with the patient’s name and bed number, wrapped with surgical foreskin, and placed in an ice bucket for clinical use.

#### Transplantation of BMMNCs

Under digital subtraction angiography, a femoral artery puncture cannula was inserted into the proper hepatic artery to observe intrahepatic vascular conditions and space-occupying lesions. About 10 mL of the spare cell suspensions were then slowly injected through the left and right hepatic arteries within 3 h. After extubation, the puncture site was bandaged with pressure. The lower extremity on the side of puncture remained immobilized for 6 h. Transplantation should be performed within 6 h after cells isolation and purification.

### Follow up

All patients were followed by reviewing their inpatient or outpatient medical records and conducting telephone interviews. The last follow-up date was November 6, 2023. Specific dates of death during follow-up were requested as well as liver transplantation, if any.

### Statistical analyses

PSM was employed to identify the patients assigned to the BMMNCs group and non-BMMNCs group. Continuous data were expressed as mean ± standard deviation and median (range), and compared using the student *t* tests or nonparametric Mann-Whitney *U* test. Categorical data were expressed as frequency (percentage) and compared with the chi-squared or Fisher’s exact tests. Cox regression and competing risk analyses were performed to explore the predictors of death in cirrhotic patients. Hazard ratio (HR) and sub-distribution hazard ratio (sHR) and their 95% confidence intervals were calculated. Multivariate Cox regression analyses and competing risk analyses were performed by adjusting for age, sex, and Child-Pugh score to explore whether BMMNCs transplantation was an independent predictor of death. Competing event for death was liver transplantation. Kaplan-Meier curve and Nelson-Aalen cumulative risk curve analyses were used to evaluate the cumulative rates of death, and compared by the logrank tests and Gray’s tests, respectively. Subgroup analyses were performed to explore the impact of BMMNCs transplantation on the prognosis of cirrhotic patients according to the age, sex, Child-Pugh class, and MELD score. A two-tailed *P* < 0.05 was considered statistically significant. All statistical analyses were performed with Stata/ SE 12.0 (Stata Corp, College Station, TX, USA) software, IBM Statistical Package for the Social Sciences (SPSS) version 27.0 (IBM Corp, Armonk, New York, USA), and R version 4.2.2 (R Foundation for Statistical Computing, Vienna, Austria) with packages ggplot2, survival, survminer, cmprsk and forestplot.

## Results

### Patient characteristics

**Figure 1:**
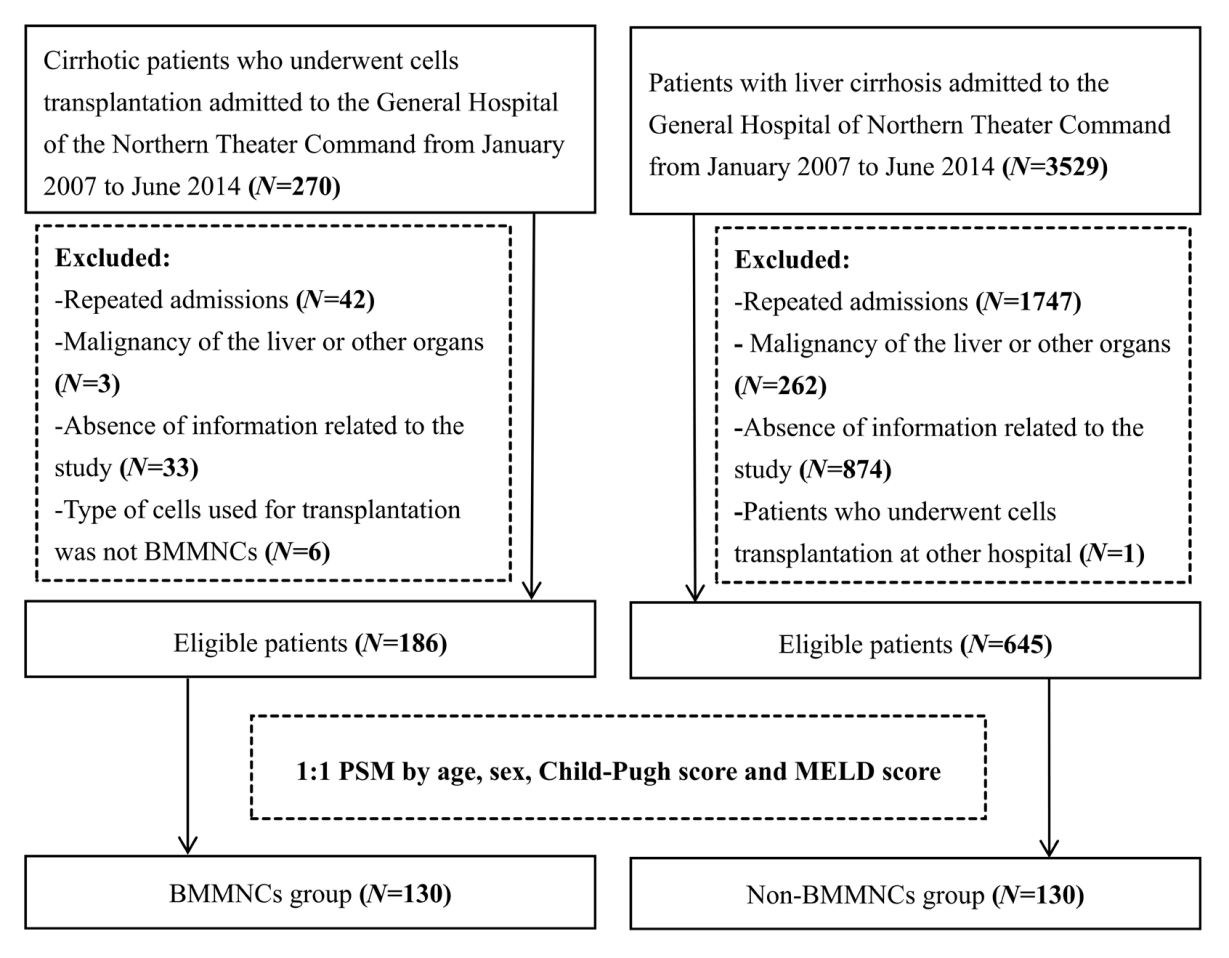
Flowchart of patients’ screening and grouping. BMMNCs: bone marrow mononuclear cells; PSM, propensity score matching; MELD: model for end-stage liver disease.

Overall, 831 patients were potentially eligible, of whom 186 received conventional therapy in combination with BMMNCs transplantation and 645 received conventional therapy alone. After PSM, 130 patients were included in each group (Figure 1).

Baseline characteristics of the patients are summarized in Table 1. Their mean age was 53.27 ± 11.12 years, and 172 (66.2%) patients were male. The most common etiology of liver cirrhosis was hepatitis B virus infection (43.1%). The mean MELD and Child-Pugh scores were 12.07 ± 5.28 and 7.60 ± 2.02, respectively.

**Table 1:** Characteristics of included patients.

	Overall group (*N*=260)		BMMNCs group (*N*=130)		Non-BMMNCs group (*N*=130)		
Variables	No. Pts	Mean ± SD, Median (range) or Frequency (percentage)	No. Pts	Mean ± SD, Median (range) or Frequency (percentage)	No. Pts	Mean ± SD, Median (range) or Frequency (percentage)	*P* Value
Demographics							
Age (years)	260	53.27 ± 11.12	130	52.96 ± 10.97	130	53.58 ± 11.30	0.652
		53.78 (20.10-82.68)		54.04 (25.85-82.68)		53.29 (20.10-78.39)	
Male (%)	260	172 (66.2%)	130	85 (65.4%)	130	87 (66.9%)	0.793
Etiology of liver cirrhosis							
HBV infection alone (%)	260	112 (43.1%)	130	77 (59.2%)	130	35 (26.9%)	<0.001
HCV infection alone (%)	260	25 (9.6%)	130	14 (10.8%)	130	11 (8.5%)	0.528
Alcohol-related liver disease alone (%)	260	66 (25.4%)	130	16 (12.3%)	130	50 (38.5%)	<0.001
Complications at admission							
Ascites (%)	260	153 (58.8%)	130	76 (58.5%)	130	77 (59.2%)	0.900
HE (%)	260	17 (6.5%)	130	8 (6.2%)	130	9 (6.9%)	0.802
GIB (%)	260	60 (23.1%)	130	18 (13.8%)	130	42 (32.3%)	<0.001
Laboratory parameters							
		97.88 ± 27.69		103.71 ± 25.70		92.10 ± 28.47	
HB (g/L)	259	98.00 (31.00-165.00)	129	104.00 (44.00-161.00)	130	92.00 (31.00-165.00)	<0.001
WBC (10^9^/L)	259	4.95 ± 3.21	129	4.91 ± 3.30	130	5.00 ± 3.14	0.530
		4.20 (1.20-24.70)		4.10 (1.20-19.20)		4.40 (1.20-24.70)	
		91.64 ± 64.93		88.53 ± 57.76		94.73 ± 71.44	
PLT (10^9^/L)	259	74.00 (17.00-464.00)	129	71.00 (21.00-320.00)	130	80.50 (17.00-464.00)	0.526
		34.04 ± 37.31		32.56 ± 32.97		35.53 ± 41.28	
TBIL (μmol/L)	260	22.20 (3.80-372.10)	130	24.35 (5.00-332.60)	130	21.50 (3.80-372.10)	0.400
ALB (g/L)	260	32.72 ± 6.93	130	34.14 ± 6.82	130	31.31 ± 6.76	<0.001
		32.85 (12.40-52.10)		34.30 (15.40-52.10)		31.10 (12.40-45.90)	
		63.06 ± 94.46		63.43 ± 100.89		62.78 ± 87.94	
AST (U/L)	260	41.00 (9.00-1079.00)	130	42.50 (10.00-1079.00)	130	39.00 (9.00-819.00)	0.322
		49.99 ± 107.65					
ALT (U/L)	260	30.50 (6.00-1460.00)	130	47.88 ± 70.60 34.00 (9.00-756.00)	130	52.10 ± 135.18 27.00 (6.00-1460.00)	0.013
		109.99 ± 82.66		107.73 ± 89.95		112.26 ± 74.95	
AKP (U/L)	260	86.50 (34.00-631.00)	130	83.50 (35.00-631.00)	130	88.85 (34.00-466.60)	0.286
		79.66 ± 95.11		86.95 ± 107.96		72.38 ± 80.00	
Scr (μmol/L)	260	60.00 (30.00-904.00)	130	65.00 (30.00-904.00)	130	57.00 (30.00-742.00)	0.005
		139.19 ± 4.00		139.95 ± 3.50		138.44 ± 4.32	
Na (mmol/L)	258	139.70 (122.70-147.70)	128	140.60 (129.40-146.80)	130	139.35 (122.70-147.70)	0.004
INR	260	1.32 ± 0.38	130	1.30 ± 0.35	130	1.34 ± 0.41	0.380
		1.22 (0.82-3.64)		1.20 (0.88-2.49)		1.23 (0.82-3.64)	
		7.60 ± 2.02		7.40 ± 2.06		7.81 ± 1.96	
Child-Pugh score	260	7.00 (5.00-13.00)	130	7.00 (5.00-13.00)	130	8.00 (5.00-13.00)	0.085
Child-Pugh class							
A (%)	260	81 (31.2%)	130	47 (36.2%)	130	34 (26.2%)	0.082
B (%)	260	136 (52.3%)	130	61 (46.9%)	130	75 (57.7%)	0.082
C (%)	260	43 (16.5%)	130	22 (16.9%)	130	21 (16.2%)	0.867
		12.07 ± 5.28		11.69 ± 4.76		12.45 ± 5.74	
MELD score	260	10.59 (2.39-31.57)	130	10.69 (2.39-27.17)	130	10.50 (6.43-31.57)	0.650

ALB, albumin; AST, aspartate aminotransferase; ALT, alanine aminotransferase; AKP, alkaline phosphatase; BMMNCs, bone marrow mononuclear cells; GIB, gastrointestinal bleeding; HBV, hepatitis B virus; HCV, hepatitis C virus; HE, hepatic encephalopathy; HB, hemoglobin; INR, international normalized ratio; MELD, model of end-stage liver disease; No. Pts, number of patients; Na, serum sodium; PLT, platelet count; SD, standard deviation; Scr, serum creatinine; TBIL, total bilirubin; WBC, white blood cell.

The proportion of male (65.4% *vs*. 66.9%, *P* = 0.793), age (52.96 ± 10.97 years *vs*. 53.58 ± 11.30 years, *P* = 0.652), MELD score (11.69 ± 4.76 *vs*. 12.45 ± 5.74, *P* = 0.650), and Child-Pugh score (7.40 ± 2.06 *vs*. 7.81 ± 1.96, *P* = 0.085) were not significantly different between BMMNCs group and non-BMMNCs group. BMMNCs group had significantly higher Hb level (103.71 ± 25.70 g/L *vs*. 92.10 ± 28.47 g/L, *P* < 0.001) and ALB level (34.14 ± 6.82 g/L *vs*. 31.31 ± 6.76 g/L, *P* < 0.001), but a lower proportion of GIB (13.8% *vs*. 32.3%, *P* < 0.001) than non-BMMNCs group (Table 1).

### Overall analysis

During a median follow-up period of 5.27 years (range: 0.37–16.62 years), 5 (1.9%) underwent liver transplantation and 130 (50.0%) died.

Univariate Cox regression analysis demonstrated that BMMNCs transplantation was significantly associated with a lower risk of death in cirrhotic patients (HR = 0.648, 95% CI = 0.484–0.868, *P* = 0.004). Multivariate Cox regression analysis demonstrated that BMMNCs transplantation was still independently associated with a lower risk of death (HR = 0.707, 95%CI = 0.528–0.946, *P* = 0.020).

Univariate competing risk analysis demonstrated that BMMNCs transplantation was significantly associated with a lower risk of death in cirrhotic patients (sHR = 0.652, 95%CI = 0.488–0.871, *P* = 0.004). Multivariate competing risk analysis also showed that BMMNCs transplantation was significantly associated with a lower risk of mortality (sHR = 0.709, 95%CI = 0.524–0.959, *P* = 0.026).

**Figure 2:**
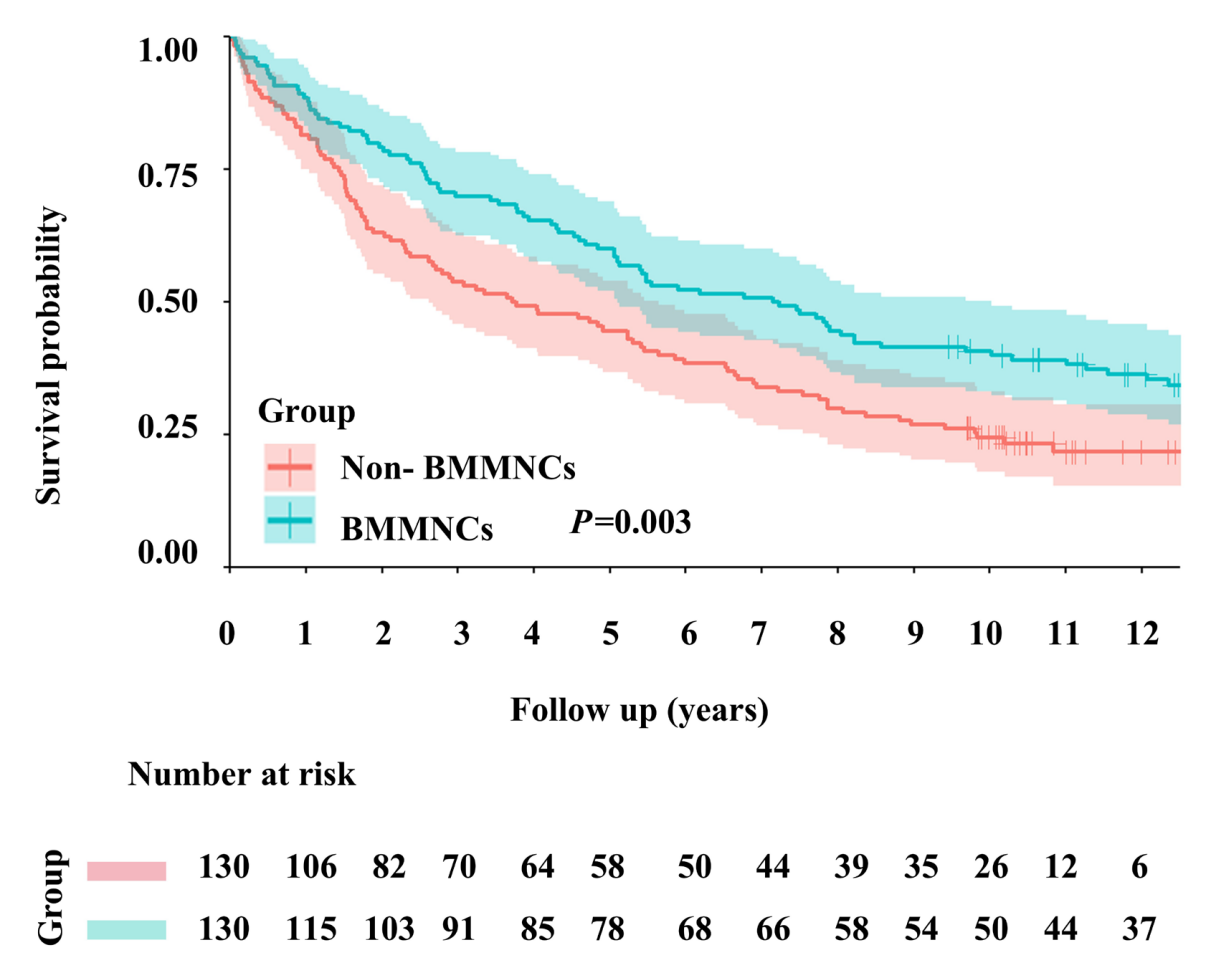
Kaplan-Meier curve analyses demonstrating the cumulative rates of survival in patients with liver cirrhosis. BMMNCs: bone marrow mononuclear cells.

The 1-, 3-, 5-, and 10-year cumulative survival rates were 88.5%, 70.0%, 59.2%, and 39.9% in the BMMNCs group and 80.8%, 53.1%, 44.6%, and 24.5% in the non-BMMNCs group. Kaplan-Meier curve analysis demonstrated that BMMNCs group had a significantly higher cumulative survival rate than non-BMMNCs group (*P* = 0.003) (Figure 2).

**Figure 3:**
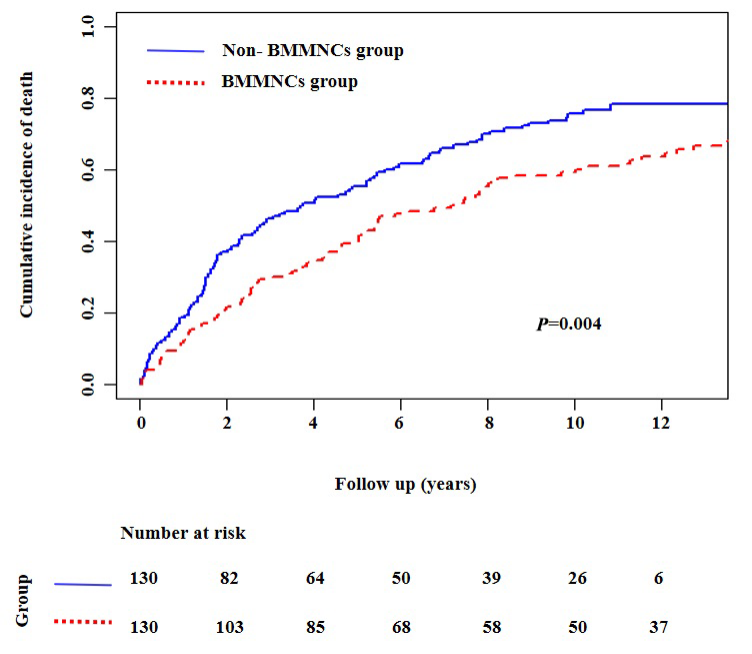
Nelson-Aalen cumulative risk curve analyses showing the cumulative mortality in patients with liver cirrhosis. BMMNCs: bone marrow mononuclear cells.

Nelson-Aalen cumulative risk curve analysis also demonstrated that the cumulative incidence of death was significantly lower in BMMNCs group than non-BMMNCs group (*P* = 0.004) (Figure 3).

### Subgroup analyses

**Figure 4:**
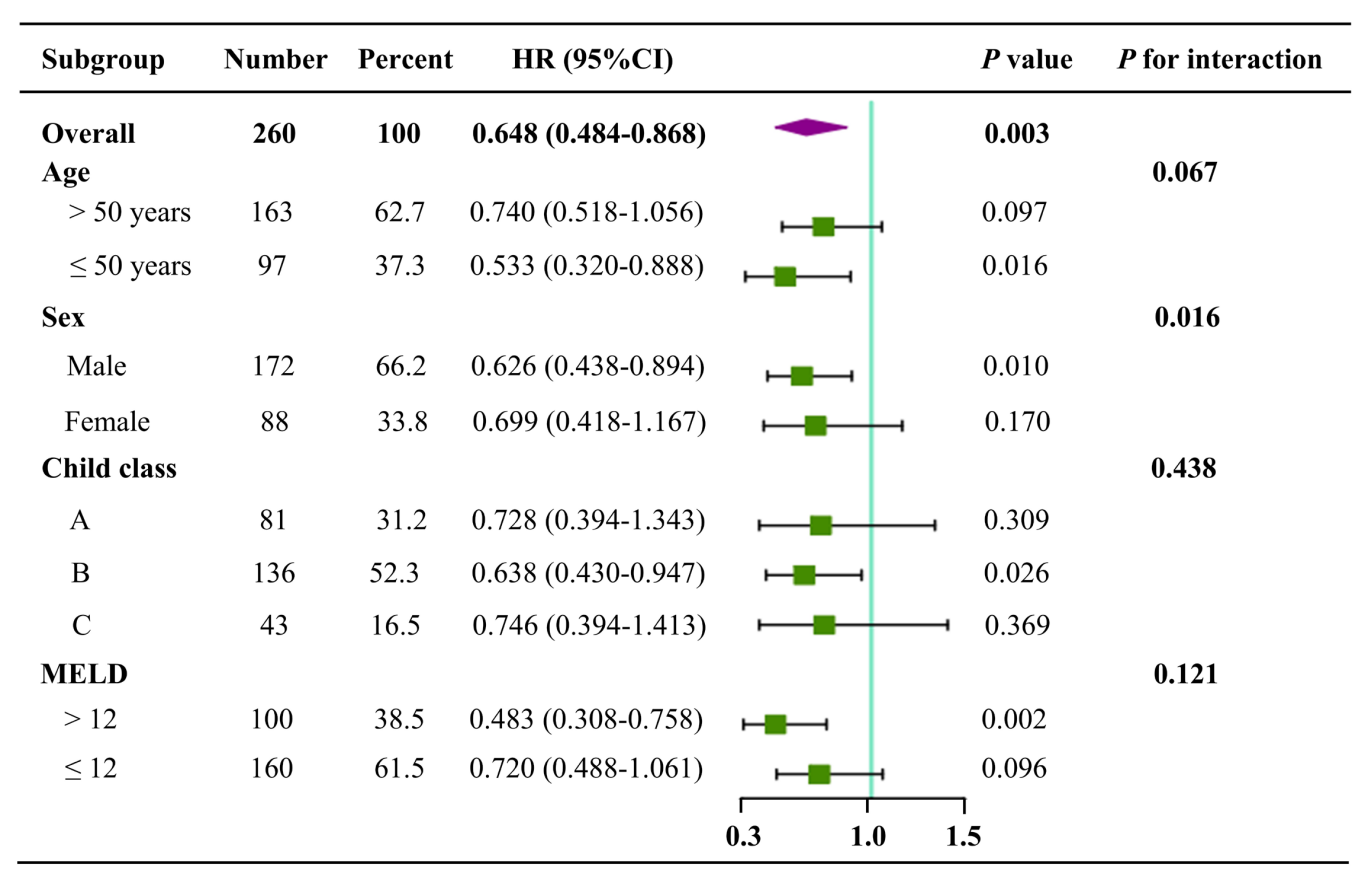
Forest plots of subgroup analyses according to age, sex, Child-Pugh class, and MELD score. HR, hazard ratio; CI, confidence interval.

**Age**: Univariate Cox regression analyses showed that BMMNCs transplantation could significantly decrease the mortality in cirrhotic patients ≤50 years (HR = 0.533, 95%CI = 0.320–0.888, *P* = 0.016), but not those > 50 years (HR = 0.740, 95%CI = 0.518–1.056, *P* = 0.097) (Figure 4).

**Sex**: Univariate Cox regression analyses showed that BMMNCs transplantation could significantly decrease the mortality in male patients with liver cirrhosis (HR = 0.626, 95%CI = 0.438–0.894, *P* = 0.010), but not female patients (HR = 0.699, 95%CI = 0.418–1.167, *P* = 0.170) (Figure 4).

**Child-Pugh class**: Univariate Cox regression analyses showed that BMMNCs transplantation could significantly decrease the mortality in cirrhotic patients with Child-Pugh class B (HR = 0.638, 95%CI = 0.430–0.947, *P* = 0.026), but not those with Child-Pugh class A (HR = 0.728, 95%C I= 0.394–1.343, *P* = 0.309) and C (HR = 0.746, 95%CI = 0.394–1.413, *P* = 0.369) (Figure 4).

**MELD score**: Univariate Cox regression analyses showed that BMMNCs transplantation could significantly decrease the mortality in cirrhotic patients with MELD score of > 12 (HR = 0.483, 95%CI = 0.308–0.758, *P* = 0.002), but not those with MELD score of ≤12 (HR = 0.720, 95%CI = 0.488–1.061, *P* = 0.096) (Figure 4).

## Discussion

The main finding of our study is that transhepatic arterial transplantation of BMMNCs can significantly improve the prognosis of cirrhotic patients. Additionally, the benefits of BMMNCs transplantation were more pronounced in cirrhotic patients who were ≤50 years and male and had Child-Pugh class B and MELD score of > 12.

Autologous BMMNCs are a promising alternative for the treatment of advanced liver diseases. However, only short-term follow-up outcomes of BMMNCs transplantation have been reported in most of relevant studies; by comparison, the data regarding long-term prognosis of BMMNCs transplantation in cirrhosis is greatly insufficient.^[23,28]^ Kim *et al*. conducted a single-arm clinical study showing that transplantation of BMMNCs is safe and can improve liver function and survival in patients with liver diseases.^[29]^ Baldo *et al*. established an animal model to investigate the therapeutic effect of BMMNCs transplantation on acute liver injury induced by carbon tetrachloride (CCl_4_) in rats, and found that BMMNCs transplantation can improve the survival and induce hepatocyte proliferation in rats.^[30]^ Belardinelli *et al*. also conducted an animal study showing that adult-derived BMMNCs significantly improved survival in a model of acute liver failure induced by acetaminophen in rats.^[31]^ In contrast to previous studies regarding bone marrow-derived cells for the treatment of liver disease (Table 2),^[22,28,29, 32, 33, 34, 35, 36, 37, 38, 39, 40, 41, 42, 43, 44]^ our study not only conducted up to 16 years of follow-up, but also employed the PSM method to minimize baseline differences between the two groups, and our patients received conventional therapy combined with a single transhepatic arterial infusion of BMMNCs. Most importantly, our study had an unprecedented follow-up period of up to 16 years after transplantation of BMMNCs in cirrhotic patients and confirmed a significant survival benefit over the follow-up period. Thus, our study provides novel evidence to support the use of BMMNCs transplantation for improving the long-term prognosis of liver cirrhosis.

**Table 2:** Summary of clinical studies of BMCs therapy for liver cirrhosis.

First author (year)	Country	Liver disease	Sample size (BMCs/Control)	Average age (BMCs/Control)	Cell dose	Times of injection	Administration route	Follow-up period (weeks)
Pai^[44]^ (2008)	United Kingdom	Liver cirrhosis	9	53	2.3×10^8^cells/patient	Single	Hepatic artery	12
Salama^[32]^ (2010)	Egypt	End-stage liver cirrhosis	90/50	50.3/50.9	0.5×10^8^cells/patient	Single	Portal vein	24
Peng^[40]^ (2011)	China	HBV-related liver failure	6/15	42.19/42.22	(3.4±3.8)× 10^8^ cells/patient	Single	Hepatic artery	192
Mohamadnejad^[33]^ (2013)	Iran	Decompensated liver cirrhosis	14/11	43.1/34.6	(1.2–2.95)×10^8^ cells/patient	Single	Peripheral vein	48
Spahr^[34]^ (2013)	Switzerland	Alcohol-related liver disease	28/30	54.0/56.0	(0.47±0.15)× 10^8^ cells/kg	Single	Hepatic artery	12
Park^[43]^ (2013)	Korea	Liver failure	5	44	NA	Single	Hepatic artery	16
Xu^[36]^ (2014)	China	HBV-related liver cirrhosis	20/19	44.0/45.0	(8.45±3.28)× 10^8^ cells/patient	Single	Hepatic artery	24
Salama^[35]^ (2014)	Egypt	HCV-related liver disease	20/20	50.27/50.9	1×10^6^cells/kg	Single	Peripheral vein	26
Bai^[22]^ (2014)	China	Decompensated liver cirrhosis	32/15	46.4/47.4	NA	Single	Hepatic artery	96
Kantarcıoğlu ^[42]^ (2015)	Turkey	Liver cirrhosis	12	39	1×10^6^cells/kg	Single	Peripheral vein	48
Suk^[38]^ (2016)	South Korea	Alcohol-related liver disease	37/18	53.8/53.7	5×10^7^cells/patient	Single/Multiple	Hepatic artery	48
Mohamadnejad^[37]^ (2016)		Decompensated liver cirrhosis			(7.62±5.53)×10^8^ cells/patient			
	Iran		10/9	43.9/46.2	(9.17±5.24)×10^8^ cells/patient	Multiple	Portal vein	48
Kim^[29]^ (2017)	South Korea	Decompensated liver cirrhosis	19	52	0.925×10^8^cells/kg	Single	Peripheral vein	264
Zhang^[39]^ (2017)	China	Liver fibrosis	30/30	31.0/32.1	6×10^6^cells/patient	Multiple	Peripheral vein	12
Lin^[41]^ (2017)	China	Acute-on-chronic liver failure	56/54	40.0/42.8	(1-10)×10^5^cells/kg	Multiple	Peripheral vein	24
Schacher^[28]^ (2021)	Brazil	Acute-on-chronic liver failure	4/5	55.8/53.4	1×10^6^cells/kg	Multiple	Peripheral vein	13

BMCs, bone marrow-derived cells; HCV, hepatitis C virus; HBV, hepatitis B virus.

In addition, our study also specifies target populations that would derive the most benefit from BMMNCs transplantation. The benefits of BMMNCs transplantation were more pronounced in cirrhotic patients who were ≤50 years and male and had Child-Pugh class B and MELD score of > 12. First, bone marrow monocytes should be more abundant and of higher quality in younger individuals, and the telomerase activity in stem cells should also be higher, which can maintain stronger self-renewal and differentiation ability. In addition, there are higher levels of stromal cell-derived factor 1 in younger individuals, which promotes BMMNCs homing.^[45,46]^ Second, male patients have high testosterone content, which can up-regulate the expression of epidermal growth factor receptor in hepatocytes by activating androgen receptors to enhance the epidermal growth factor response secreted by BMMNCs.^[47]^ Third, Child-Pugh class B and MELD score > 12 indicated moderate liver dysfunction, where hepatic regeneration signals can be more readily activated, thereby promoting the migration of BMMNCs to the injured area of the liver. Meanwhile, in the setting of moderate liver dysfunction, the remaining normal hepatocytes can also respond to these regenerative signals, thus contributing to the paracrine effect of BMMNCs. By comparison, in patients with severe liver dysfunction, hepatic regeneration is remarkably inhibited, compromising the effect of BMMNCs on regulation of liver microenvironment and promotion of liver tissue repair.^[48, 49, 50]^

**Figure 5:**
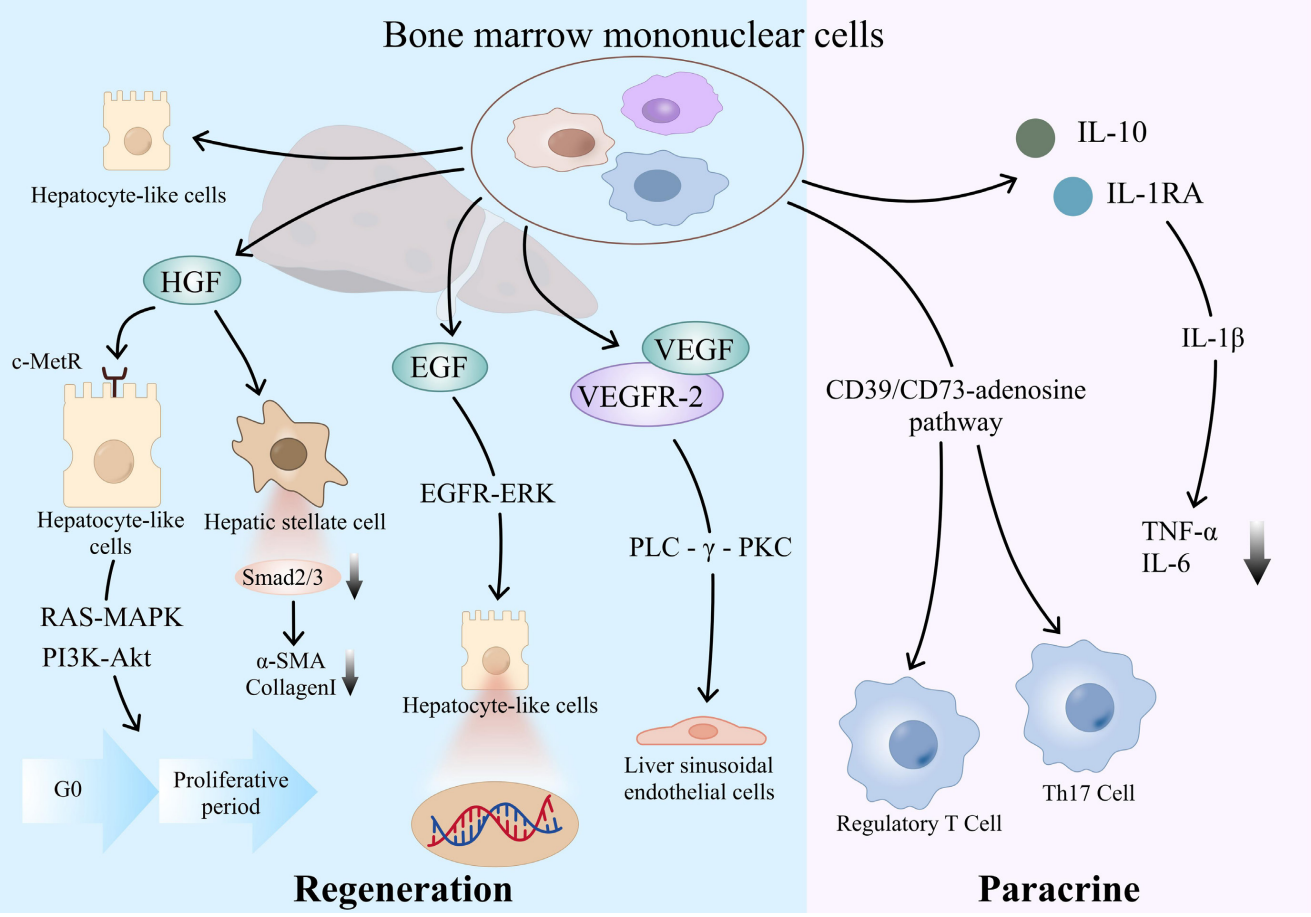
Mechanism diagram of action of BMMNCs. BMMNCs: bone marrow mononuclear cells.

BMMNCs are known for their paracrine and regenerative promoting properties, which may play a crucial role in the regulation of liver microenvironment. First, BMMNCs is a heterogeneous cell population containing a variety of progenitor cells, in which mesenchymal stem cells and hematopoietic stem cells can be induced by the liver injury microenvironment to differentiate into hepatocyte-like cells *via* the Wnt/β-catenin and Notch pathways and directly supplement functional hepatocytes.^[51]^ It can also release a variety of growth factors, such as HGF, epidermal growth factor, and VEGF. HGF can bind to the c-Met receptor on the surface of liver cell membrane, activate the Ras-MAPK and PI3K-Akt pathways, induce the expression of cyclin, and drive hepatocytes to enter the proliferation cycle from the stationary G0 phase. Moreover, the mediation of TGF-β1 on α-SMA and collagen I expression was blocked by inhibiting Smad2/3 phosphorylation in hepatic stellate cells, and matrix metalloproteinases were upregulated to promote fiber degradation.^[18,52]^ Epidermal growth factor enhances DNA synthesis in hepatocytes through EGFR-ERK pathway, forming a synergistic effect with HGF, which can improve the liver regeneration.^[53]^ VEGF activates the PLC-gamma-PKC pathway by binding to VEGFR-2, stimulates the proliferation of hepatic sinusoid endothelial cells, increases functional capillary density, reduces the level of hypoxia-inducing factor-1α in liver, reduces hypoxia-driven inflammatory factors, such as IL-6 and TNF-α, increases oxygen supply to damaged liver tissue, and accelerates the repair of damaged liver tissue.^[54]^ Second, BMMNCs can secrete IL-10 and IL-1RA, which competitively block IL-1β signaling, thereby reducing the levels of pro-inflammatory cytokines. Additionally, through the CD39/CD73^-^adenosine pathway, BMMNCs activate regulatory T cells, but suppresses Th17 cell differentiation, which contributes to the restoration of immune homeostasis, facilitates the establishment of immune tolerance, and ultimately attenuates inflammatory responses (Figure 5).^[15,16]^ In addition, animal experiments have shown that BMMNCs can secrete anti-inflammatory proteins and reduce oxidative stress, thus promoting liver function recovery and improving the survival in rats.^[55,56]^ Thus, stem cells should be effective for controlling inflammation. It has been shown that autologous BMMNCs transplantation seems to have good short-term efficacy in patients with viral hepatitis related liver failure.^[57]^ Similarly, a high proportion of our study population had liver cirrhosis caused by viral hepatitis, which may be one of important causes for favorable prognosis in the BMMNCs group.

In our study, a single transhepatic arterial infusion of BMMNCs was employed and G-CSF was used for bone marrow mobilization prior to cell extraction. Notably, multiple meta-analyses have shown that a single injection, transhepatic arterial infusion, and the use of bone marrow as the source of cells should be the optimal strategies for treatment of liver diseases.^[58, 59, 60]^ This may be an important advantage of our study. First, transhepatic arterial transplantation can improve cells homing ability, which is a key prerequisite for BMMNCs to play a therapeutic role in patients with cirrhosis.^[61, 62, 63]^ Yu *et al*. also indicated that enhancing cells survival rate and homing ability can augment the engraftment efficacy of cells.^[64,65]^ Second, studies have shown that the use of G-CSF before transplantation can promote homing of BMMNCs and accelerate recovery and improve survival after liver injury by promoting endogenous repair programs.^[66,67]^ Third, previous studies showed no significant difference in the improvement of liver function between patients who received multiple injections and those who received a single injection, suggesting that the number of BMMNCs injections may not affect the patients’ outcomes. Moreover, multiple transhepatic arterial transfusions carry a higher risk of vascular damage, thrombosis, and infection as compared to a single infusion.^[58]^

There are some limitations in our study. First, since this is a retrospective cohort study, the presence of selection bias and recall bias is often inevitable, which may affect the reliability of our findings to some extent. Regardless, PSM was employed to avoid these biases. Second, this study focused on the patients’ death during follow-up period. However, the specific cause of death, changes of liver function indicators, and development of decompensation events could not be provided in all patients.

In summary, BMMNCs transplantation serves as a viable alternative for cirrhotic patients who are unable to undergo liver transplantation, due to its benefits in the improvement of survival and the quality of life. In order to clarify the impact of BMMNCs transplantation on the long-term prognosis of patients with cirrhosis, further large-scale prospective studies with additional end points are needed to comprehensively evaluate the short-term and long-term prognosis of patients, so as to provide more solid evidence for clinical practice.
